# Primary care management post gestational diabetes in Australia

**DOI:** 10.1111/imj.16106

**Published:** 2023-06-22

**Authors:** Simone Marschner, N. Wah Cheung, Edwina Wing‐Lun, Samia Kazi, Ritu Trivedi, Clara K. Chow

**Affiliations:** ^1^ Westmead Applied Research Centre The University of Sydney Sydney New South Wales Australia; ^2^ Department of Diabetes & Endocrinology Westmead Hospital Sydney New South Wales Australia; ^3^ Royal Darwin Hospital, Menzies School of Health Research University of Sydney Sydney New South Wales Australia; ^4^ Department of Cardiology Westmead Hospital Sydney New South Wales Australia

**Keywords:** gestational diabetes, diabetes, pregnancy, cardiovascular disease, screening

## Abstract

**Background:**

Women with a history of gestational diabetes (GD) have a high risk of developing diabetes and subsequent cardiovascular disease (CVD).

**Aim:**

To assess whether diabetes screening and CVD risk screening occurred in general practice (GP) among postpartum women with GD.

**Methods:**

This is a retrospective study of clinical record data of women with GD, under active GP management, from the MedicineInsight programme, run by Australia's National Prescribing Service MedicineWise, with GP sites located in Australia from January 2015 to March 2021. Documentation of screening for diabetes, assessment of lipids and measurement of blood pressure (BP) was assessed using proportions and mixed‐effects logistic regression with a log follow‐up time offset.

**Results:**

There were 10 413 women, with a mean age of 37.9 years (standard deviation, 7.6), from 406 clinics with a mean follow‐up of 4.6 years (interquartile range, 1.8–6.2 years) A total of 29.41% (3062/10 413; 95% confidence interval [CI], 28.53–30.28) had not been assessed for diabetes, 37.40% (3894/10 413; 95% CI, 36.47–38.32) were not assessed for lipids and 2.19% (228/10 413; 95% CI, 1.91–2.47) had no BP documented. In total, 51.82% (5396/10 413; 95% CI, 50.86–52.78) were screened for all three (diabetes + lipids + BP) at least once. Obesity, comorbidities and dyslipidaemia were associated with increased likelihood of screening. New diabetes diagnosis was documented in 5.73% (597/10 413; 95% CI, 5.29–6.18) of the cohort.

**Conclusion:**

Screening for diabetes and hyperlipidaemia was suboptimal in this high‐risk cohort of women with prior GD. Improved messaging that women with a GD diagnosis are at high cardiovascular risk may improve subsequent screening.

## Introduction

Cardiovascular disease (CVD) affects an estimated 275.2 million women worldwide, causes 8.94 million deaths per year^1^ and is the leading cause of death globally, being responsible for 35% of total deaths in women in 2019.^1^ Similarly in Australia, over half a million women were affected by CVD and it is the leading cause of death, being the cause for 16% of total deaths in women.^2^ Diabetes is a leading cause of CVD, with almost 1 in 20 Australian women with self‐reported type 2 diabetes,^3^ contributing to 10.5% of all deaths. Previous systematic reviews have shown that the estimated risk of type 2 diabetes is six to 10 times higher if a woman is known to have had gestational diabetes (GD).^4^, ^5^ A systematic review and meta‐analysis of six studies calculated that up to a third of parous women with diabetes would have experienced a GD pregnancy earlier.^6^ Guidelines (2020) from the Royal Australian College of General Practitioners (RACGP) for follow‐up of patients with a history of GD recommend that fasting blood glucose and glycated haemoglobin (HbA_1c_) should be tested every 3 years and women contemplating another pregnancy should have an oral glucose tolerance test performed annually.^7^ The Australasian Diabetes in Pregnancy Society (ADIPS) states that diabetes testing should be performed every 1 to 2 years among women with normal glucose tolerance.^8^

A systematic review and meta‐analysis has shown that women with GD, compared with those without, are at a twofold increased risk of cardiovascular events, and the incidence of type 2 diabetes did not affect this association.^9^ The high risk of future diabetes and subsequent raised cardiovascular risk following complications of pregnancy has informed the American College of Cardiology and the American Heart Association guidelines to incorporate screening for diabetes in those with a history of GD.^10^

Australia's National Prescribing Service (NPS) MedicineWise was established in 1998 as a large‐scale general practice (GP) database of longitudinal deidentified electronic health records. It is funded by the Australian Government to facilitate the design, development, implementation and evaluation of national programmes for the improvement of medicine use in primary care.^11^ We utilised this valuable resource with the primary objective of assessing whether women with a history of GD have had 1 or 2 yearly regular screenings for diabetes as recommended by ADIPS^8^ and whether cardiovascular risk factors, namely blood pressure (BP) and lipids, are being assessed.

## Methods

### Data source

The MedicineInsight^11^ programme run by NPS MedicineWise, contains electronic health records from GP sites, located in every Australian state and territory. As of October 2018, MedicineInsight had recruited 662 participating GP sites across Australia, representing approximately 8.2% of all GP sites in Australia.^11^ MedicineWise provided data on all patients older than 18 years, with at least one visit to a GP from 1 January 2015 to 1 March 2021. For the purposes of the current study, we extracted information on the cohort of women with a documented diagnosis of GD based on MedicineWise coding algorithms, incorporating information from three electronic health record fields: diagnosis, the reason for the visit and the reason for a prescription.^12^ The following terms were used to identify records for inclusion:‐DIABETES ‐GESTATIONAL‐DIABETES MELLITUS‐GESTATIONAL‐DIABETES MELLITUS, GESTATIONAL‐GESTATIONAL DIABETES‐GESTATIONAL DIABETES MELLITUS. Records identified by a free text string alone were individually reviewed by a clinical coder to check the context. We included only women under active management by a GP, defined as having three or more clinical visits to a GP in the 2 years prior to the most recent visit, and those women with a GD diagnosis date after their first clinical encounter date to ensure that we only included women who were managed by their current GP.

### Statistical analysis

We estimated the proportion of patients having any documentation of diabetes screening tests, BP measurements and lipid profiles post GD. To address the factors associated with screening for diabetes in women with GD, a logistic regression model was used to analyse the binary outcome of screening (yes/no), with random effect for GP clinic and a fixed effect for age and number of clinical encounters. Differences in duration of follow‐up were adjusted for by including an offset of the log of the follow‐up time (GD diagnosis to last clinical visit or diabetes diagnosis). The following covariates were explored in this model: smoking status, quintiles of the Index of Relative Socio‐economic Disadvantage, quintiles of the Index of Economic Resources, remoteness, indigenous status, last reported body mass index (BMI), Bice–Boxerman index for continuity of care^13^ and number of comorbidities and types of medical conditions.

The number of tests for diabetes conducted over the GP engagement period was estimated overall and for each year. The GP engagement period was defined as the GD diagnosis date to their last clinical encounter. If a woman was diagnosed with diabetes, then follow‐up was censored at the date of diagnosis. A Poisson regression of the number of diabetes tests was used to assess the factors associated with the screening rate. This model included an offset of the log of the follow‐up time (GD diagnosis to last clinical visit or diabetes diagnosis) with a random effect for GP clinic adjusting for age and the number of clinical encounters. The same covariates mentioned for the other models were explored for an association with screening frequency.

To assess whether women with GD were being assessed for cardiovascular risk factors, the above‐mentioned logistic regression was used on the binary outcomes of having lipids measured (yes/no) and having BP measured (yes/no). The risk of diabetes among this cohort of GD women was estimated and the factors associated with this risk were assessed using the above‐mentioned logistic regression model. The glmer function within the package lme4 package in R^14^ was used for random‐effects logistic regression and Poisson regression analyses.

### Definitions

The Bice–Boxerman index for continuity of care is a measure of the extent that a patient is loyal to the one clinician within a clinic, which may change their health management.^13^ The number of comorbidities was categorised into four groups: GD alone, one comorbidity and two or more comorbidities. The list of comorbidities counted is shown in the supplementary table S1. Pathology records were searched for documentation of all glucose tests (glucose tolerance test [GTT], oral GTT, fasting and nonfasting glucose tests) and HbA_1c_ tests (excluding cerebrospinal fluid glucose tests and any reference to pregnancy glucose tests) as indicators for diabetes screening. For those with a documented diagnosis of diabetes, it was assumed that a diabetes screening test had been undertaken. To establish whether women were tested for BP or lipids, the pathology and clinical records were searched for any of these assessments post‐GD diagnosis until their last clinical visit or until diabetes diagnosis.

The NPS records a date for the diagnosis of GD, but not the delivery date. Our window for the first year was from GD diagnosis date plus 26 weeks to this point plus 1 year. Most GD testing is performed between 22 and 28 weeks, so by taking the midpoint of 26 weeks and adding another 26 weeks brings the start of our window to 52 weeks, namely 12 weeks post delivery date. This avoids capturing the 6‐ to 12‐week GTT, which is designed to detect persistent or even preexisting diabetes, rather than long‐term screening. Another reason for not including the 6‐ to 12‐week GTT is that this is frequently done by hospital pathology units as part of the episode of GD care, and therefore not captured in our data set, so it may not reflect the true rate of 6‐ to 12‐week testing. For this reason, it is not included in the analysis. For completeness, we also present the proportion of women who had a GTT from GD diagnosis to 26 weeks, which is presumed to capture the postpartum test. Women only contributed to each time period if their last clinical encounter was beyond the end date of that time period.

## Results

There were 10 526 actively GP‐managed women with a GD diagnosis after their first clinical encounter with a GP and their last clinical encounter at least 26 weeks after their GD diagnosis. Among these women, 37.41% (3938/10 526; 95% confidence interval [CI], 36.49–38.34) had a GTT within 26 weeks of their GD diagnosis. There were 1.07% (113/10 526) diagnosed with diabetes in this time frame and these were excluded from the primary analysis as they would not be further tested. This resulted in a cohort of 10 413 women from 406 clinics with a mean time between GD diagnosis and last clinical visit of 4.6 years (interquartile range, 1.8–6.2). Table 1 describes the cohort of women with GD with a mean age of 38 ± 7.6 years at the time of data extraction and a mean BMI of 30.6 ± 11.0 at the most recent measurement. During the follow‐up period, from 26 weeks after GD diagnosis, 29.41% (3062/10 413; 95% CI, 28.53–30.28) of women were not assessed for diabetes, 37.40% (3894/10 413; 95% CI, 36.47–38.32) of women were not tested for elevated lipids and 2.19% (228/10 413; 95% CI, 1.91–2.47) did not have BP testing documented. Only 51.82% (5396/10 413; 95% CI, 50.86–52.78) of women had all three tests performed, namely, diabetes screening, lipids assessessment and BP measurement.

**Table 1:** Characteristics of women with GD

	Total (*N* = 10 413)
Age, mean (SD)	37.9 (±7.6)
Remoteness (missing = 47, 0.5%)	
Inner regional	2131/10 366 (20.56%)
Major cities	7226/10 366 (69.71%)
Outer regional	904/10 366 (8.72%)
Remote	69/10 366 (0.67%)
Very remote	47/10 366 (0.45%)
Indigenous status (missing = 1520, 14.6%)	
Indigenous	383/8893 (4.31%)
Nonindigenous	8510/8893 (95.69%)
Index of relative socioeconomic disadvantage (missing = 47, 0.5%)	
1 = Most disadvantaged	1723/10 366 (16.62%)
2	1772/10 366 (17.09%)
3	2275/10 366 (21.95%)
4	2376/10 366 (22.92%)
5 = Most advantaged	2220/10 366 (21.42%)
Quintile index of economic resources (missing = 47, 0.5%)	
1 = Most disadvantaged	2157/10 366 (20.81%)
2	1833/10 366 (17.68%)
3	2332/10 366 (22.50%)
4	2340/10 366 (22.57%)
5 = Most advantaged	1704/10 366 (16.44%)
Smoking (missing *n* = 496, 4.8%)	
Current smoker	933/9917 (9.41%)
Ex‐smoker	1888/9917 (19.04%)
Nonsmoker	7096/9917 (71.55%)
Most recent BMI, mean (SD)	30.6 (±11.0)
Comorbidity count	
0	5501/10 413 (52.83%)
1	2328/10 413 (22.36%)
2	1357/10 413 (13.03%)
3 or more	1227/10 413 (11.78%)
Atrial fibrillation	31/10 413 (0.30%)
Coronary heart disease	49/10 413 (0.47%)
Chronic kidney disease	14/10 413 (0.13%)
Dyslipidaemia	1108/10 413 (10.64%)
Hypertension	1230/10 413 (11.81%)
Heart failure	27/10 413 (0.26%)
Stroke	76/10 413 (0.73%)
Transient ischaemic attack	72/10 413 (0.70%)
Polycystic ovarian syndrome	1055/10 413 (10.13%)

BMI, body mass index; GD, gestational diabetes; SD, standard deviation.

**Figure 1:**
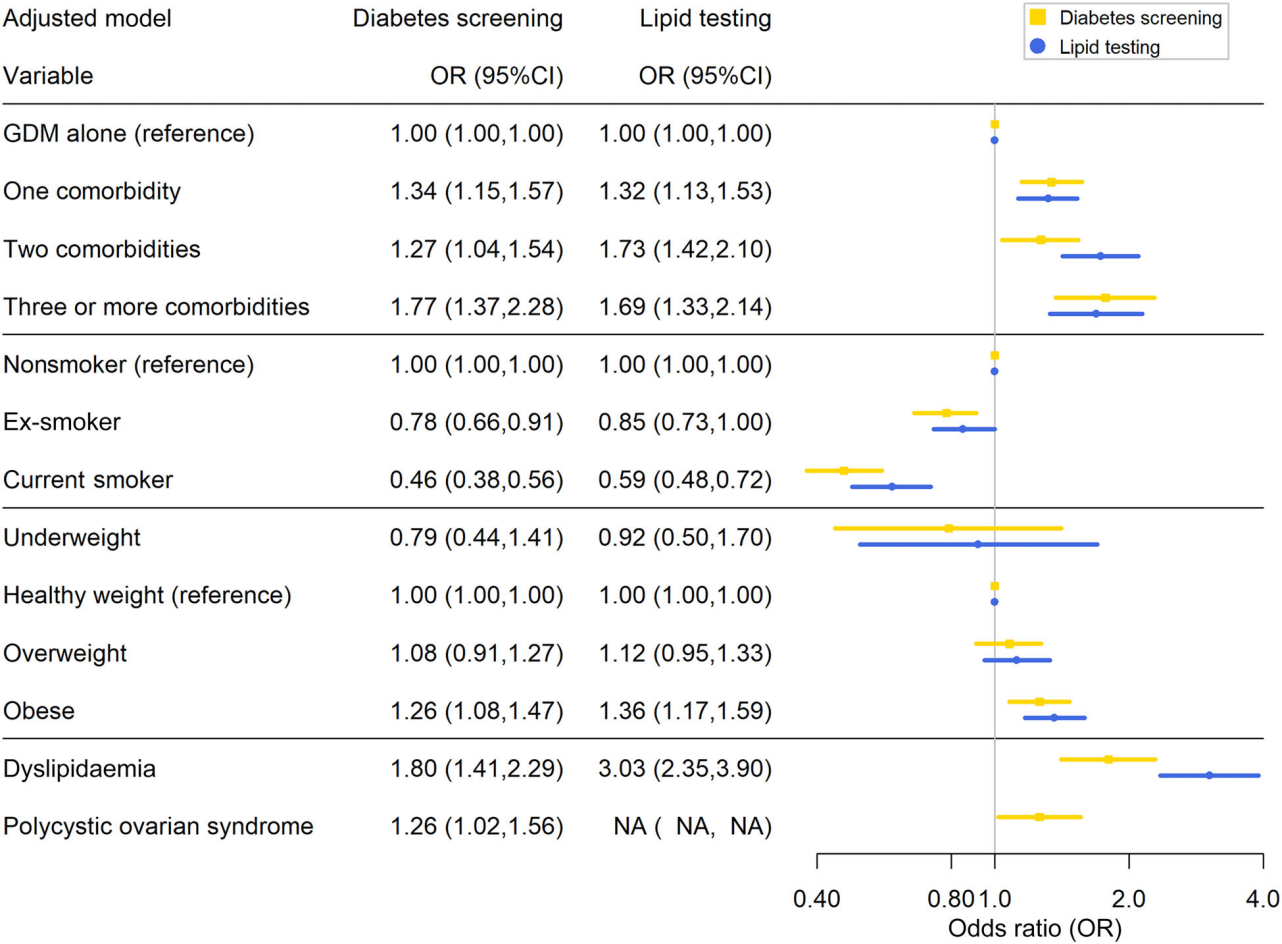
The association between risk factors and being screened for diabetes and tested for elevated lipids. CI, confidence interval; GDM, gestational diabetes; NA, not applicable.

Smoking status, BMI category and number and type of comorbidities were significantly associated with reported screening for diabetes (Fig. 1). Current smokers had lower diabetes screening rates compared with nonsmokers (odds ratio [OR], 0.46 [95% CI, 0.38–0.56]), women with obesity had higher diabetes screening rates compared with women with healthy weight (OR, 1.26 [95% CI, 1.08–1.47]) and those with three or more comorbidities (in addition to GD) were more likely to be screened than those with only GD (OR, 1.77 [95% CI, 1.37–2.28]). Furthermore, dyslipidaemia (OR, 1.80 [95% CI, 1.41–2.29]) and polycystic ovarian syndrome (OR, 1.26 [95% CI, 1.02–1.56]) were both associated with increased odds of screen testing for diabetes, even after accounting for the total number of comorbidities in the adjusted models. Similarly higher BMI, more comorbidities, having dyslipidaemia and not smoking were found to be significantly associated with increased odds of lipid testing. The directions of the associations were consistent with the results for diabetes screening. Only hypertension was significantly associated with higher BP testing (OR, 6.7 [95% CI, 2.4–18.6]) in the adjusted model.

**Figure 2:**
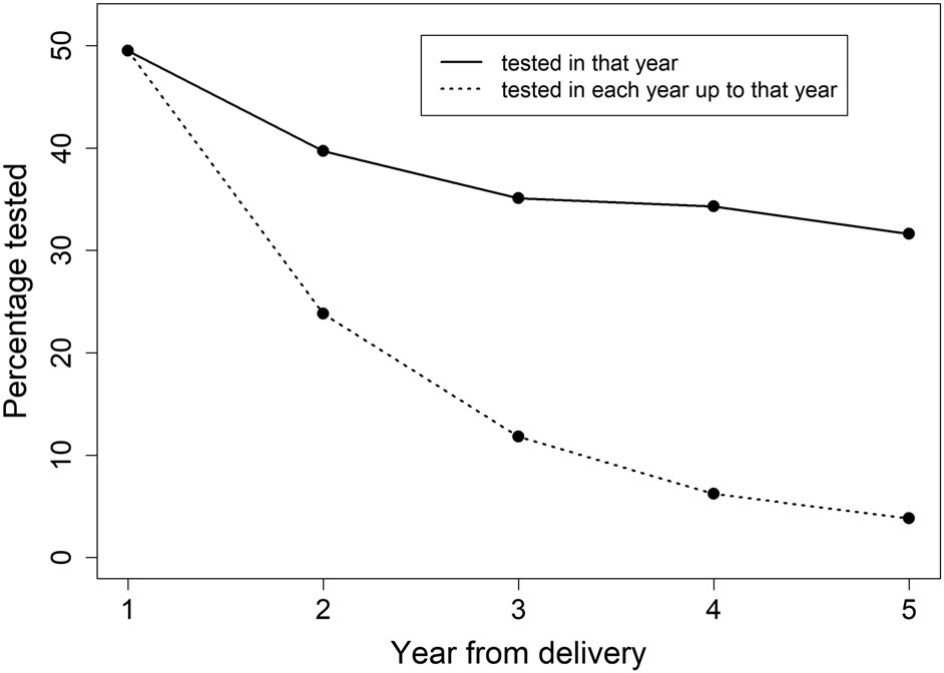
Diabetes testing post delivery by year. (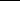) Tested in that year; (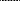) tested in each year up to that year.

In total, 49.49% (4129/8343; 95% CI, 48.42–50.56) of women had a diabetes test within their first year beyond their postpartum testing period, and the proportion tested in each subsequent year and regularly tested for diabetes declined over time (Fig. 2). For example, 39.72% (2607/6563; 95% CI, 38.54–40.91) of women were tested in the second year after delivery, whereas only 23.83% (1564/6563; 95% CI, 22.80–24.86) were tested in both the first and second years after delivery. Only 3.78% (117/3098; 95% CI, 3.11–4.45) of women were tested for diabetes every year for the first 5 years after delivery.

**Figure 3:**
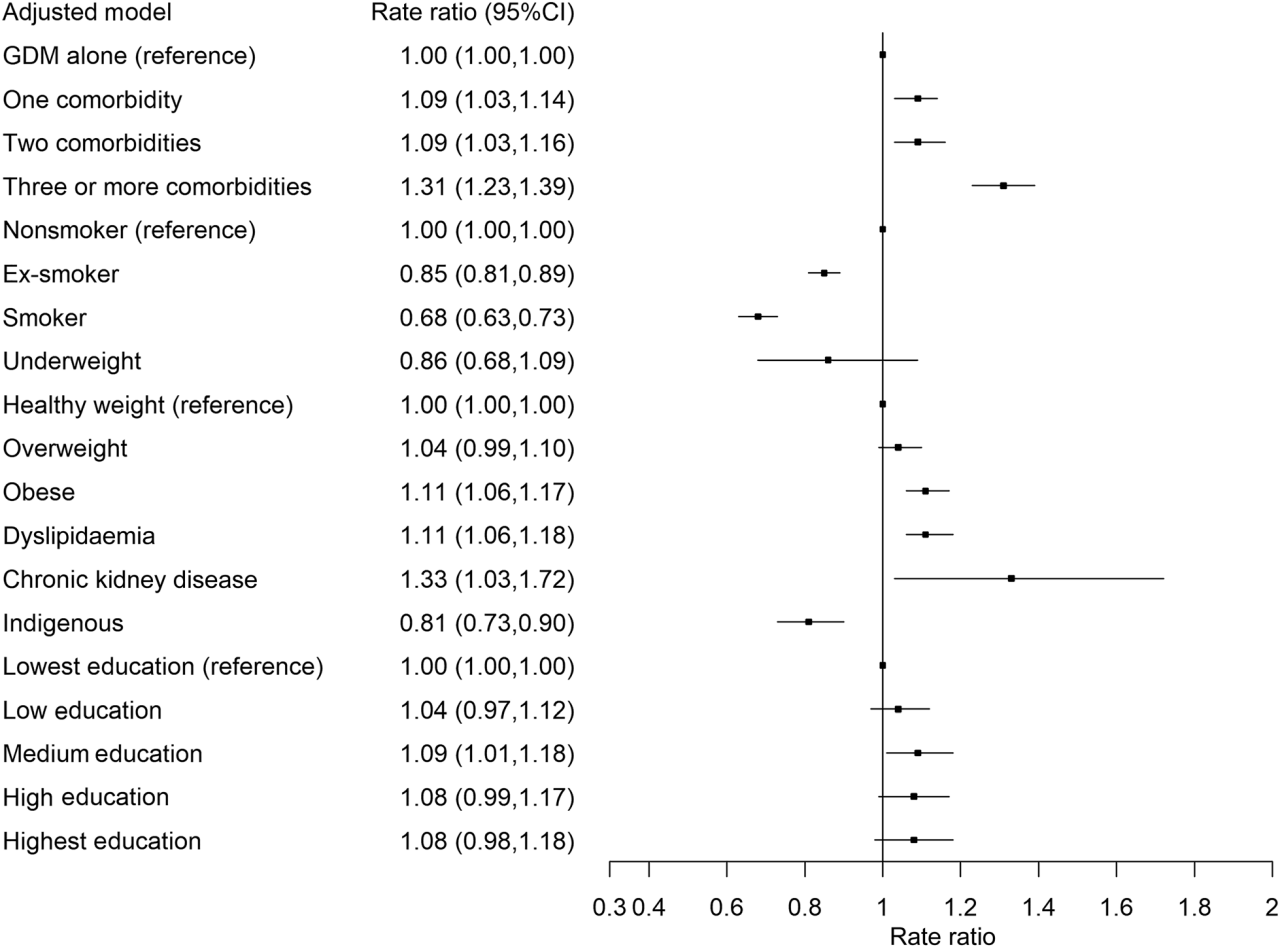
Rate ratio of glucose tests adjusted for patient characteristics. CI, confidence interval; GDM, gestational diabetes.

The rate of diabetes screening was estimated to be 0.41 tests *per annum* (95% CI, 0.37–0.46), which is equivalent to one test every 2.44 years. We found that the diabetes screening rate was lower among women with few comorbidities, lower BMI, indigenous status, smoking or lower education levels. The patient‐specific screening rate was estimated to be the overall average screening rate, modified according to patient characteristics, using the rate ratios shown in Figure 3. For example, a woman with obesity and four comorbidities has a 45% (1.11 × 1.31 = 1.45) higher testing rate than a woman of healthy weight and only GD, assuming identical values for all other risk factors.

**Figure 4:**
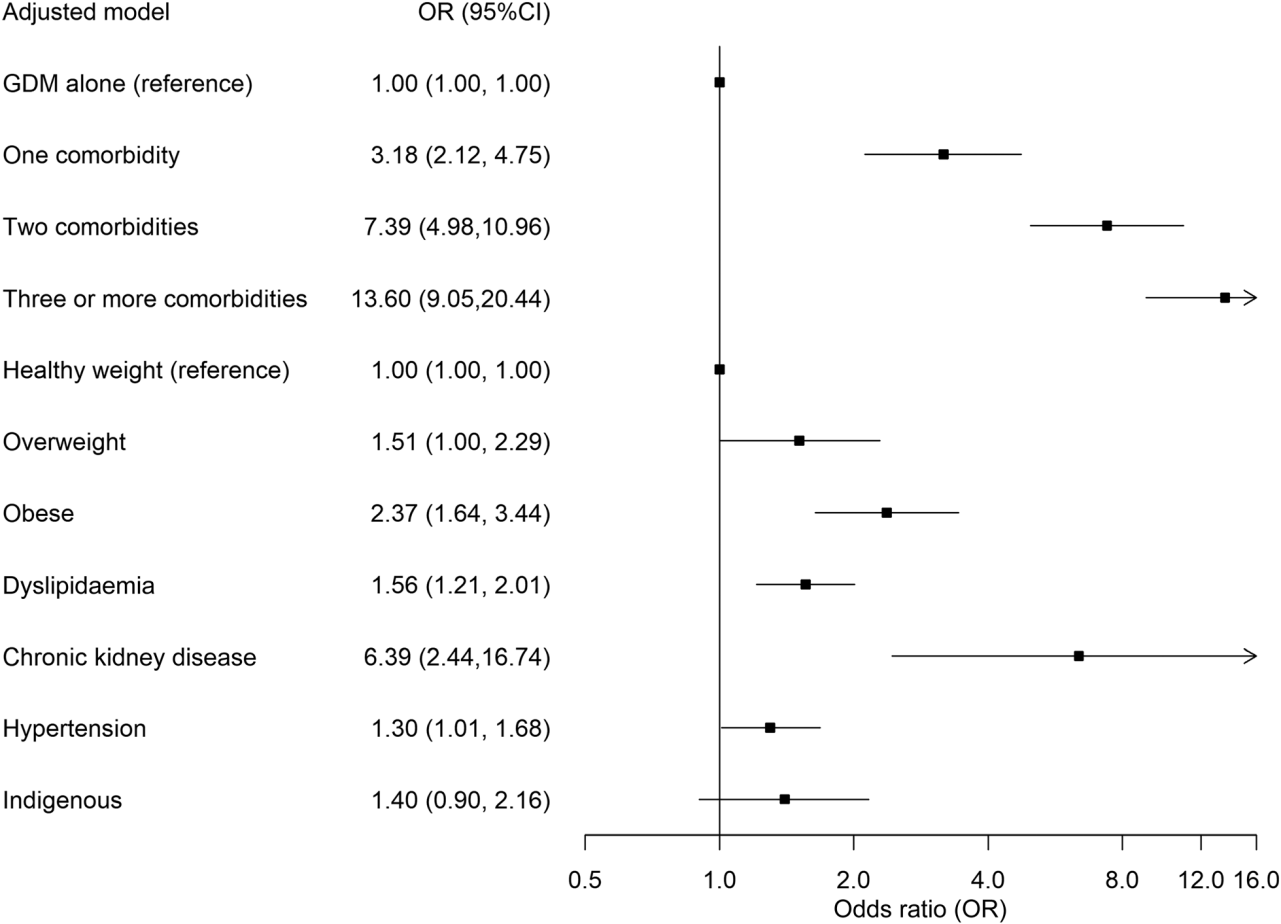
Factors associated with a diagnosis of diabetes in an adjusted model. CI, confidence interval; GDM, gestational diabetes.

New diabetes diagnosis after GD occurred in 5.73% (597/10 413; 95% CI, 5.29–6.18) of the cohort. Factors associated with increased risk of diabetes diagnosis in this cohort of women with GD were more comorbidities, higher BMI, dyslipidaemia, chronic kidney disease, indigenous status and hypertension (Fig. 4).

## Discussion

We found that among women who had a history of GD, and therefore at known high risk of future diabetes, who had regularly visited a GP, over one‐quarter (29.41%, 95% CI, 28.53–30.28) had no documentation of having been tested for diabetes beyond postpartum testing over a median follow‐up of 4.6 years. Over one‐third (37.38%, 95% CI, 36.45–38.30) had postpartum diabetes testing. Over one‐third of women had no evidence of lipid testing (37.40%, 95% CI, 36.47–38.32), 2.19% (95% CI, 1.91–2.47) had no documentation that BP was measured and only half had all three tests performed (51.82%, 95% CI, 50.86–52.78). Obesity, more comorbidities and dyslipidaemia were significantly associated with the occurrence of testing for glucose, lipids and BP among women with GD, suggesting that GPs were less likely to screen for CVD risk if women only had GD and more likely to screen if additional risk factors were also present.

A retrospective cohort study of 10 868 women with GD found that a suboptimal number (under a quarter [23.9%]) of women received annual testing^15^ as recommended by UK National Institute for Health and Care Excellence (NICE) guidelines.^16^ We estimated that our cohort obtained one test every 2.44 years, which is consistent with Australian RACGP recommendations of once every 3 years, but is less frequent than is recommended by NICE and ADIPS guidelines of 1 to 2 years.^7^, ^8^ Our promising estimate may partly be explained by the selection of an actively managed cohort and also by the National Diabetes Services Scheme (NDSS) sending out reminders to women registered on the NDSS with GD and their GPs.^17^

A systematic review and meta‐analysis of postpartum diabetes screening has documented rates of screening for postpartum diabetes averaging 35.0% for studies up to 3 months and 36.5% for studies up to 6 months,^18^ which is very similar to our result of 37.4% and less than ideal. Our result, however, is likely to be an underestimate as postpartum testing is often undertaken by hospital pathology and we did not have access to this data.

Monitoring lipids among patients with type 2 diabetes in rural Australia found that 30.6% did not obtain their annual lipid tests.^19^ Our findings are consistent with these results and indicate that despite guidelines and persistent public health efforts to highlight the need for diabetes testing and cardiovascular risk screening among people at risk, the rate of testing has further to improve in Australian primary care. The higher levels of BP measurement, compared with other risk factors, could be because BP testing is simple and accessible, sphygmomanometers are readily available and GP guidelines encourage routine BP measurements. Other potential barriers are concerns about reimbursement given guidelines differ in recommended frequency and the need to fast for lipid profiles.

Obesity, hypertension and dyslipidaemia are strongly associated with diabetes, as comorbidities, which form the metabolic syndrome.^20^, ^21^ These are also factors that are independently associated with CVD.^21^ These known associations may explain our findings of increased testing among women with obesity and those with dyslipidaemia; however, hypertension was not as strongly associated with diabetes screening and lipid testing. Contrary also to this notion is that in this study, current smokers were less likely to have tests performed. One possible explanation for this observation is that smokers are less inclined to see their clinicians or follow through on tests that have been ordered. This behaviour has been observed among smokers with lung cancer symptoms.^22^

Despite our cohort being young women (mean age, 38 years), with a recent GD diagnosis (median follow‐up, ~5 years) and screening rates suboptimal, we still found a higher rate of subsequent diabetes diagnosed (5.73%) compared with the population prevalence of ~1.4% for women in this age group in Australia,^23^ supporting prior evidence that GD places women at high risk of diabetes. The International Association of Diabetes and Pregnancy Study Groups diagnosis criteria was accepted by the Australasian Diabetes in Pregnancy Society in 2014, reducing the cutoff criteria for GD diagnosis. Most women in our cohort were diagnosed after this criterion change, which therefore included more women with ‘mild’ GD. Despite this, our GD cohort still has a four times higher rate of subsequent diabetes than the general population. Our rates are lower than an Australian cohort study reporting a 10.3% prevalence rate of diabetes after GD^24^; however, in this study, most women were diagnosed prior to 2014. Even with our current‐day lower diagnosis cutoff of GD, a diagnosis of GD still indicates that these women are at high risk for diabetes. Consistent with other research, we found that the number of comorbidities, BMI, dyslipidaemia, chronic kidney disease, indigenous status and hypertension were all associated with increased risk of diabetes following GD.^25^

A limitation of this analysis is that we only examined pathology results from each patient's GP visits within the database. As patients could be comanaged by other GPs or specialists, there may have been additional testing so our results may underestimate the frequency of testing. It is also possible that some of the glucose testing that we interpreted as testing for diabetes, were in fact performed during subsequent pregnancies as testing for GD. We minimised this limitation by excluding glucose tests tagged as occurring during pregnancy, but tests may not have been all accurately tagged. There were challenges defining the time window of 1‐year post delivery without a record of the actual date of delivery. We undertook a cautious conservative approach, which may have excluded diabetes testing immediately post partum.

## Conclusion

In a world that is increasingly realising that inadequate emphasis is placed on CVD by women, health care and health system providers, there is a need to identify approaches to better improve CVD prevention in women. Identifying at‐risk women early, at a point where their future risk is modifiable through screening for diabetes and cardiovascular risk among women with GD, is one such potential approach. Better messaging to improve awareness, electronic decision support in primary care, reminders and incentivisation of women post partum are all possible interventions^26^ that could help prevent the longer‐term risks of diabetes and CVD.

## Supporting information


**Table S1:** Each co‐morbidity in the first column were counted in the total number of co‐morbidity count variable. They were considered present if any of the flags (generated from MedicineInsight) in the second column was present.
