# Supplementary material for: Primary care management post gestational diabetes in Australia
Source: Intern Med J. 2023 Jun 22;54(1):164–71. doi: 10.1111/imj.16106 (PMC10952553; doi:10.1111/imj.16106)
Supplement: Supplementary file 1 — Table S1: Each co‐morbidity in the first column were counted in the total number of co‐morbidity count variable. They were considered present if any of the flags (generated from MedicineInsight) in the second column was present. [file IMJ-54-164-s001.docx]

SUPPLEMENT TABLE

S1: Each co-morbidity in the first column were counted in the total number of co-morbidity count variable. They were considered present if any of the flags (generated from MedicineInsight) in the second column was present.

| Atrial fibrillation/flutter | f_AF; f_AFL |
| --- | --- |
| Atherosclerotic disease | f_CASTEN; f_CASTEN_PR; f_CHD_ATH; f_CHD_ATH_PR; f_PVD; f_RASTEN; f_RASTEN_PR |
| Heart failure | f_HF |
| Rheumatic heart disease | f_RHEUHEAR |
| Stroke | f_str_all; f_STR_H; f_STR_I; f_STR_L; f_STR_M; f_STR_T; f_STR_US; f_TIA |
| Dyslipidaemia | f_DYS; f_HYPERC; f_HYPERLI; f_HYPERTRIG |
| Hypertension | f_HYPT |
| Diabetes | f_DM_T1; f_DM_T2; f_DM_US; f_DM_T3 |
| Polycystic ovarian syndrome | f_PCOS |
| Thyroid disorders | f_THYROID_HYPER; f_THYROID_HYPO; f_THYROID_UNSPEC |
| Chronic kidney disease | f_CKD_3; f_CKD_4; f_CKD_5; f_CKD_UNSP |
| Cancer | f_CANC |
| Arthritis | f_ARTH |
| Chronic pain | f_PAIN_CHR; f_PAIN_BACK_L |
| Osteoarthritis | f_OSTEO |
| Osteoporosis | f_OP |
| Rheumatoid arthritis | f_ARTH_RH; f_ARTH_JRA |
| Anxiety and depression | f_ANX; f_DEPR |
| Bipolar | f_BIPOL |
| Dementia | f_DEMEN |
| Epilepsy | f_EPIL |
| Schizophrenia | f_SCHIZ |
| Substance abuse | f_ABU_SUB |
| Asthma | f_ASTH |
| COPD | f_COPD |
| Chronic Liver Disease | f_CLD |
| Coeliac Disease | f_COELIAC |
| Crohn's Disease | f_CROHNS |
| Ulcerative Colitis | f_COLI_ULC |
